# Evaluation of Aerodynamic Drag and Torque for External Tanks in Low Earth Orbit

**DOI:** 10.6028/jres.111.014

**Published:** 2006-04-01

**Authors:** William C. Stone, Christoph Witzgall

**Affiliations:** National Institute of Standards and Technology, Gaithersburg, MD 20899

**Keywords:** aerodynamic drag, aerodynamic eccentricity, aerodynamic torque, free molecular flow theory, low Earth orbit, shell of revolution, space shuttle external tank

## Abstract

A numerical procedure is described in which the aerodynamic drag and torque in low Earth orbit are calculated for a prototype Space Shuttle external tank and its components, the “LO2” and “LH2” tanks, carrying liquid oxygen and hydrogen, respectively, for any given angle of attack. Calculations assume the hypersonic limit of free molecular flow theory. Each shell of revolution is assumed to be described by a series of parametric equations for their respective contours. It is discretized into circular cross sections perpendicular to the axis of revolution, which yield a series of ellipses when projected according to the given angle of attack. The drag profile, that is, the projection of the entire shell is approximated by the convex envelope of those ellipses. The area of the drag profile, that is, the drag area, and its center of area moment, that is, the drag center, are then calculated and permit determination of the drag vector and the eccentricity vector from the center of gravity of the shell to the drag center. The aerodynamic torque is obtained as the cross product of those vectors. The tanks are assumed to be either evacuated or pressurized with a uniform internal gas distribution: dynamic shifting of the tank center of mass due to residual propellant sloshing is not considered.

## Introduction

During the intervening 15 years since the first publication of this paper,^1^ a great many things have changed in the space business. Of the original introduction that follows, many things anticipated then did not come to pass. The international space station is clinging for survival with less than half its intended crew, 24 years after its design was begun. Orbital makeup due to atmospheric drag on that large structure remains an ever-present problem now that access is limited to a few Russian Soyuz flights a year. The cost of delivering a kilogram of load to Low Earth Orbit (LEO) via the space shuttle was recently assessed at $30,000 when considering the full amortization of the standing army required to prepare it for launch. The failure of the shuttle system to reduce costs of transport to LEO is now recognized. Alternative launch system approaches are being considered for the future “Crew Exploration Vehicle” (CEV). Private endeavors, such as Burt Rutan’s novel SpaceShipOne architecture which recently captured the Ansari X-Prize, suggest that entrepreneurs may outpace government efforts in getting to LEO cheaply. Ironically, such private efforts need a destination to turn them into profitable tourism offerings—destinations in LEO that would represent pressurized, large volume habitable facilities–facilities not unlike those originally foreseen by many as one use for the shuttle E-tank. Yet, today there are no fleets of orbiting External Tanks—converted to Spartan industrial laboratories (or hotels)—as envisioned in this paper. Despite the inherent utility of such enormous storage facilities in orbit, that prospect today appears almost non-existent in the face of E-tank TPS shedding being identified as the initiating event in the loss of the shuttle *Columbia* in 2003. The E-tanks became collateral victims in what has become an inability to accept that exploration and work on the frontier comes with finite risk. We believe that this is unfortunate, because when one views the history of the space shuttle from its inception to its ultimate retirement, planned for 2010, the establishment of a vast orbital tank farm might have been seen as the greatest accomplishment of the shuttle era. Two important reconnaissance spacecraft, flown around the moon in 1994 (DoD’s Clementine) and 1999 (NASA’s Lunar Polar Orbiter), have since identified the presence of no less than 10 billion metric tons of hydrogen signature at the lunar south pole, probably of meteoric (asteroid) origin and most probably in the form of pulverized water ice mixed with regolith. That is enough hydrogen and oxygen to bootstrap a commodity-based positive return on Earth-moon investment that would serve as a springboard to the expansion of human exploration throughout the inner solar system. The E-tanks could have provided an economical fuel depot for that commercial product in LEO. Of the mathematics that follow, they are as pure, clean, and compact as they were in 1989, as if they had never aged, their utility beckoning.

We feel it is instructive to reproduce below the introduction to the original (1989) paper.

## 1. Introduction (1989)

Within the next ten years it is anticipated that a significant number of structures exhibiting very large drag profiles will be placed in low earth orbit (LEO). Large orbital structures are not new: the Echo I experimental inflatable satellite had a projected drag area of some 725 m^2^ [6]. What is new is that the facilities planned for the 1990’s will be at substantially lower altitude than Echo I (300 km to 500 km vs. 1000 km) and of such mass (in excess of 30 metric tons) that random re-entry cannot be permitted on grounds of safety. Significant engineering problems arise because positive control of such craft requires motors for attitude and altitude changes. For asymmetrical structures as much as an order of magnitude difference can exist in the amount of fuel required for orbit maintenance depending on the spacecraft orientation with respect to its velocity vector. This is of particular concern to entrepreneurial commercial space companies seeking financing for the placement of such structures on orbit, since it presently costs approximately $30,000 per pound in transport costs to LEO alone.

Of particular interest to the present study is the external tank of the Space Shuttle, that is, the U.S. Space Transportation System (STS). The external tank is currently the only non-reusable component of the STS. On a typical launch to an orbit inclined 28.5° with respect to the equator (a due east launch from Cape Kennedy) these tanks, which carry cryogenic oxygen and hydrogen to fuel the three main shuttle engines, reach approximately 98 % of orbital velocity at an altitude of about 100 km [8]. It is possible for the shuttle to take these tanks into relatively low orbit for only a modest penalty in terms of reduced shuttle payload capacity for most missions and at no penalty on some missions which are limited by weight and balance considerations [8]. Given that the amortized cost of taking an object of similar mass to orbit would be well in excess of $200 million, there is a compelling argument to consider making use of these tanks, rather than allowing them to re-enter the earth’s atmosphere following main engine cut-off (MECO) as is present practice. The economies of employing such “used” equipment on orbit were recognized as early as 1976 [10] and several detailed studies concerning various uses for external tanks were carried out in the early 1980’s [8,9].

An agreement between the University Corporation for Atmospheric Research (UCAR) and NASA [14], signed in December 1988 grants approval to instrument five STS external tanks for sub-orbital flights within the next three years. These sub-orbital missions will constitute tests of flight hardware eventually to be used to place external tanks in long-term stable orbits. The control of such tanks, which weigh more than 30 metric tons and which have a total exterior surface area about half that of a football field, poses a considerable challenge. Considering the potentially broad impact that the availability of such assets on orbit will have on commercial space enterprises, NIST has undertaken a program to study the problems surrounding the control and conversion of such structures to habitable facilities on orbit at the lowest possible cost while maintaining safety. The problem of controlling an earth-orbiting spacecraft may be divided into the following four components: orbit determination, attitude determination, attitude control, and altitude control. Four principal disturbing forces acting on objects in low earth orbit must be quantified in order to determine the control requirements described above. These are gravity-gradient torque, solar radiation pressure (and induced torque), aerodynamic drag (and induced torque), and magnetic disturbance torque [5]. The aerodynamic component is dominant at altitudes below 400 km and is addressed in this paper. Documentation of the computer code which underlies the numerical results reported in the paper will be found in a forthcoming technical report [16]. Determination of aerodynamic drag and torque has been investigated previously in various contexts [2,3,4].

Under the assumptions of free molecular flow theory, no boundary layer is formed. Molecules re-emitted from a surface do not collide with free stream molecules until far away from the body. One may thus neglect distortions of the free stream velocity distribution due to the presence of the body, and assume that aerodynamic drag force is entirely due to impact of atmospheric molecules on the spacecraft surface. For hypersonic flows impinging on cool surfaces, the momentum of molecules leaving the surface may be neglected. The impact of molecules in the incident stream may thus be modeled as inelastic without reflection, that is, the incident particle’s energy is completely absorbed [1]. It is also assumed that attitude (orientation) changes are slow compared to the translational velocity of the spacecraft.

## 2. Drag Area and Aerodynamic Eccentricity

Let **v** denote the unit vector in the direction of the translational velocity V of the center of gravity of the spacecraft relative to the incident stream, and let **i** be the attitude vector, that is, the unit vector in the direction of the axis of revolution oriented from rear to front of the spacecraft. The angle between the velocity vector **v** and the attitude vector **i** is the angle of attack *θ*, where:

0°<θ≤180°

**Fig. 1:**
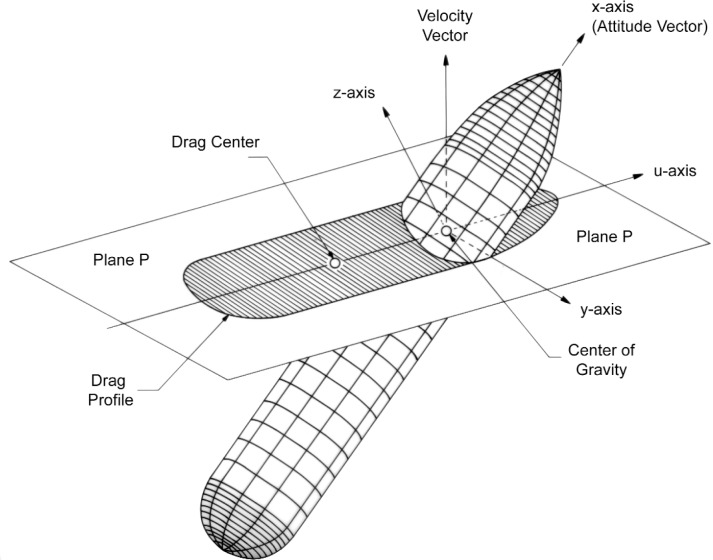
Geometry of the drag profile of a shell of revolution.

Consider the plane **P** (Fig. 1) which is perpendicular to **v** and passes through the center of gravity of the body, which is assumed to lie on the axis of revolution. The projection of the shell surface in the direction of **v** into plane **P** will be referred to as the drag profile. Due to the rotational symmetry of the surface, the shape and the size of the drag profile depend solely on the angle of attack. As we will outline below, the drag force **F**_AERO_ and the aerodynamic torque **N**_AERO_ are determined by the area A_DRAG_—referred to as the drag area–and the center of area moment–referred to as the drag center— of the drag profile.

The differential drag force *d***F**_AERO_ acting on a surface element *dA* with outward unit normal **n**_S_ is given by:

$$dF_{\mathrm{AERO}}=-\frac{1}{2}\rho C_{\mathrm{DRAG}}V^{2}(n_{s}\cdot v)vdA$$

where *ρ* is the atmospheric density [5]. Analytical models for the atmosphere up to an altitude of 110 km [11] and for altitudes above 90 km [12] are available which provide seasonal (as affected by solar activity) and latitudinal values of *ρ*. The parameter C_DRAG_ is the drag coefficient and is, in general, a function of the surface structure. In the limiting hypersonic case, only forward-facing surface elements contribute to drag and for these the value C_DRAG_ = 2.0 [7] is suggested.

Note that the factor (**n**_S_ · **v**)*dA* in the above expression for the differential drag force *d* **F**_AERO_ represents the projection of a surface element *dA* in direction **v** onto plane **P**. Now the total drag force **F**_AERO_ is obtained by integrating the contributions from all forward-facing exterior surface elements of the spacecraft, that is, those surface elements for which the product (**n**_S_ · **v**) is positive. The projections of these elements exactly cover the projection of the total surface, that is, the drag profile. In other words,

$$\int (n_{s}\cdot v)dA=A_{\mathrm{DRAG}}$$

The total drag force vector may thus be expressed as:

$$F_{\mathrm{AERO}}=-\frac{1}{2}\rho C_{\mathrm{DRAG}}V^{2}vA_{\mathrm{DRAG}}$$

The aerodynamic torque **N**_AERO_ acting on the spacecraft due to the differential force *d* **F**_AERO_ is given by the integral:

$$N_{\mathrm{AERO}}=\int R_{s}\times dF_{\mathrm{AERO}}$$

where **R**_S_ is the vector from the spacecraft’s center of gravity to the surface element *d*A. The integral is taken over the spacecraft surface for which (**n**_S_ · **v**) is positive. Substituting for the differential drag force *d***F**_AERO_ yields:

$$N_{\mathrm{AERO}}=-\frac{1}{2}\rho C_{\mathrm{DRAG}}V^{2}\int (R_{s}\times v)(n_{s}\cdot v)dA$$

In order to evaluate the integral in the above expression, we recall that (**n**_S_ · **v**)*d*A is the projection of surface element *d*A into the surface element of plane **P**. Since that plane contains the center of gravity of the body, and since the vector **R**_S_ originates at that center, the vector **R**_S_× **v** leads—within plane **P**—from the center of gravity to the projected surface element. The integral in question thus reduces in plane **P** to a familiar expression: when divided by area over which the integration extends, it indicates the location of the center of area moment of the drag profile, namely, the drag center, with respect to the common origin of vectors **R**_S_, namely, the center of gravity of the body. Denoting by **R**_CG_ the eccentricity vector which leads from the center of gravity to the drag center we thus have:

$$\int (R_{s}\times v)(n_{s}\cdot v)dA=A_{\mathrm{DRAG}}R_{\mathrm{CG}}$$

and

$$\begin{array}{c} N_{\mathrm{AERO}}=-\frac{1}{2}\rho C_{\mathrm{DRAG}}V^{2}A_{\mathrm{DRAG}}R_{\mathrm{CG}}\\ =R_{\mathrm{CG}}\times F_{\mathrm{AERO}}. \end{array}$$

The aerodynamic eccentricity is a scalar whose absolute value is the length of the eccentricity vector **R**_CG_. It is positive if the drag center leads the center of gravity in the direction of the attitude vector, which points from the rear towards the front of the spacecraft, and negative if it trails. In terms of the attitude vector **i**:

$$e_{\mathrm{AERO}}=\mathrm{sign}(R_{\mathrm{CG}}\cdot i)\left\|R_{\mathrm{CG}}\right\|$$

Due again to the rotational symmetry of the shell and because its center of gravity lies on the axis of revolution, the eccentricity depends only on the angle of attack. The remainder of this paper is devoted to the description of a numerical procedure for the determination of the drag area, A_DRAG_, and the drag center, needed to find the aerodynamic eccentricity e_AERO_ for convex shells of revolution under a specified angle of attack *θ*.

## 3. Description of Shells of Revolution

**Fig. 2:**
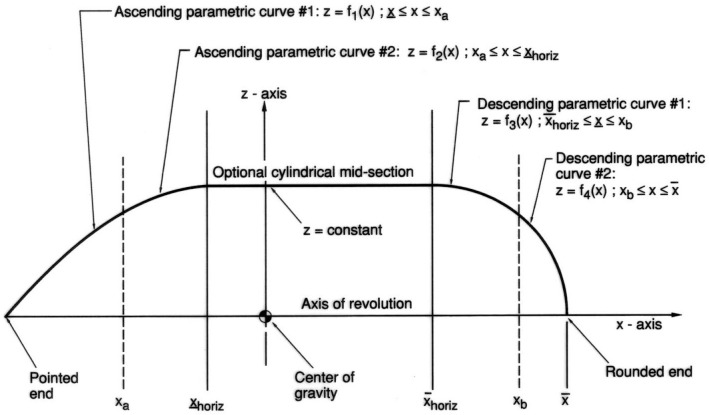
Schematic of a shell contour with cylindrical portion.

The present software implementation of our method is restricted to a particular category—to be characterized below—of convex shells of revolution. The geometric shape of the Space Shuttle external tank falls into that category. We assume the shells to be embedded in *x,y,z*-space with the x-axis in the direction of the attitude vector (see Fig. 1). Each shell is characterized by its “contour,” that is, the graph of a function,

Z=r(x)≥0,

over a closed interval $\left[\underline{x},\bar{x}\right]$ (Fig. 2). The longitudinal section of the shell in the *x, z*-plane, whose rotation sweeps the volume of the shell, is then bounded above by + *r*(*x*) and below by – *r*(*x*). Typically, the domain $\left[\underline{x},\bar{x}\right]$ will be partitioned into a series of intervals in each of which the contour function will be expressed by a suitable parametric equation.

For the shell of revolution to be convex it is necessary and sufficient that the contour function be concave. Beyond this property, we will require that the contour consists of (see Fig. 2):

1. To the left, an ascending strictly concave function with unique tangents which starts with a value of zero, $r(\underline{x})=0\;$, and terminates with a horizontal tangent, $r^{\prime }({\underline{x}}_{\mathrm{HORIZ}})=0$;
2. In the middle, an optional horizontal line at maximum function value *r*_MAX_ (corresponding to an optional cylindrical middle section of the shell) which starts at the end of the previous function, $r({\underline{x}}_{\mathrm{HORIZ}})=r_{\mathrm{MAX}}$;
3. To the right, a descending strictly concave function with unique tangents which starts at the end $r({\underline{x}}_{\mathrm{HORIZ}})=r_{\mathrm{MAX}}$ of the cylindrical portion— provided such is present—with a horizontal tangent, $r^{\prime }({\underline{x}}_{\mathrm{HORIZ}})=0$, and terminates with a value of zero, $r(\bar{x})=0$. Where ${\underline{x}}_{\mathrm{HORIZ}}\leq {\bar{x}}_{\mathrm{HORIZ}}$ denote the left and right ends of the horizontal portion; ${\underline{x}}_{\mathrm{HORIZ}}\leq {\bar{x}}_{\mathrm{HORIZ}}$ if there is none present.

By “strictly concave” it is meant that there is a continual change of the tangent direction and therefore no straight line segments in the graph of the function to which the term is applied. Thus the horizontal straight line representing the optional cylindrical middle section is the only straight line segment permitted presently in the contour. Since the above left portion of the contour function terminates at its right with a horizontal tangent and the above right portion starts with a horizontal tangent to its left, each point of the entire contour function has a unique slope. The contour function is therefore differentiable everywhere in the interior of the domain. The above partitionability of the contour function into an ascending, a descending and, optionally, a horizontal portion does not mean that only three parametric equations are permitted for the description of the contour function: any number of such equations can be used as long as the resulting function meets the above requirements.

At the endpoints $\underline{x}$ and $\bar{x}$ of the domain of the contour function, we permit infinite values for its derivatives. That is, $r^{\prime }(\underline{x})=+\infty$ and/or $r^{\prime }(\bar{x})=-\infty$ may hold, indicating vertical end tangents. In those cases, the ends ( $\underline{x}$, 0,0) and ( $\bar{x}$, 0,0), respectively, of the shell of revolution are “rounded.” They are “pointed” at those ends if the corresponding end tangents are non-vertical. For instance, the shell of revolution generated by the contour depicted in Fig. 2 has a pointed left end and a rounded right end.

For the applications considered here, the shell of revolution represents the shape of a physical object such as the Space Shuttle external tank. In this case, it will be convenient to locate the origin of the coordinate system at the center of gravity of the physical object. For the external tank, this center of gravity would be chosen for an empty tank, or a tank with a uniform pressurized internal atmosphere such as might exist following on-orbit modifications to create a shirtsleeve workshop. The effects of residual fuel sloshing and dynamically shifting the location of the center of gravity are beyond the scope of this paper.

In the previous section, a velocity vector **v** was introduced. Because of the rotational symmetry, we may assume that this vector lies in the vertical *z, x*-plane. The angle *θ* between the velocity vector **v** and the *x*-axis, or attitude vector, is the angle of attack. The vector **v** also determines the plane **P** which is perpendicular to it and contains the origin of the *x,y,z*-coordinate system, that is, the center of gravity as shown in Fig. 1. This plane contains the *y*-axis. The intersection of **P** with the *z,x*-plane yields a line that is perpendicular to the *y*-axis, and can be selected as the *u*-axis of the *u,y*-coordinate system in that plane **P**. We direct the *u*-axis so that its angle with the *x*-axis lies between 0° and 90°. The orthogonal projection, that is, the shadow cast in the direction of the velocity vector **v** by the shell of revolution onto the plane **P** is the drag profile, whose shape, area A_DRAG_, and center of area moment, the drag center (**u**_DRAG_, 0) are at issue. Since both + **v** and – **v** yield the same drag profile in the same plane **P**, we may assume without loss of generality that the angle of attack *θ* lies between 0° and 90°: 0° ≤ *θ* ≤ 90°. If 0°, then the drag profile is given by the circular cross section of largest diameter. We will therefore assume in the next two sections that *θ* is positive:≤θ

0°≤θ≤90°.

## 4. Discretization of Shells of Revolution

Our method for determining the drag profile is based on approximating the contour function *z* = *r*(*x*) in a piecewise linear fashion as follows:

$$\underline{x}=x_{1}<x_{2}<\ldots <x_{i}<\ldots <x_{N}=\bar{x}$$

from its domain $\left[\underline{x},\bar{x}\right]$, and connect adjacent points (*x_i_*, *z_i_* = *r*(*x_i_*)), (*x_i_*_+ 1_, *z_i_*_+ 1_ = *r*(*x_i_*_+ 1_)) in the graph of the contour function by straight line segments. The resulting shell of revolution consists of a sequence of slices of circular cones or of cylinders, the circular top of one forming the base for the next. The orthogonal projection of such a “piecewise conical” shell of revolution can be described in closed form as described below. A different way of looking at the same procedure is as a discretization method that approximates the given shell of revolution by a finite sequence of circular cross sections, which project a finite sequence of circular cross sections, which project into a sequence of similar and parallel ellipses. The drag profile is then approximated by the convex envelope, that is, the smallest convex set enclosing those ellipses.

The approximate drag profile is thus determined by a sequence of ellipses **E***_i_*, *i* = 1,…,*n*. The centers of these ellipses are located on the *u*-axis of plane **P** with coordinates *u_i_* = *x_i_* sin *θ*. The major axes *a_i_* = *r*(*x_i_*) are in the direction of the *y*-axis. The minor axes lie on the *u*-axis, their length given by *b_i_* = *a_i_* cos *θ*. The equation of the ellipse **E***_i_* is therefore:

$$\left(\frac{u-u_{i}}{b_{i}}\right)+\left(\frac{y}{a_{i}}\right)=1$$

The first and the last of these ellipses may be degenerate, *a_i_* = *b_i_* = 0, and consist of a single point.

If any ellipse in the above sequence is contained in a neighboring one, then such an ellipse can be deleted without changing the convex envelope. This usually happens at the beginning and the end of the sequence of ellipses. More precisely, there is a largest index $\bar{i}$ such that ellipse **E***_i_* contains all previous ellipses **E***_i_*, $i<\bar{i}$. Analogously, there is a smallest index $\underline{i}$ such that ellipse **E***_i_* contains all subsequent ellipses **E***_i_*, $i<\underline{i}$ where:

$$1\leq \underline{i}\leq \bar{i}\leq n$$

Since none of the ellipses **E**_i_ with $\underline{i}\leq i\leq \bar{i}$ contains any of the others, all that is necessary in order to delineate their convex hull is to join subsequent ellipses by their common tangents. We should clarify that we mean those common tangents which have the ellipses on equal rather than different sides.

**Fig. 3:**
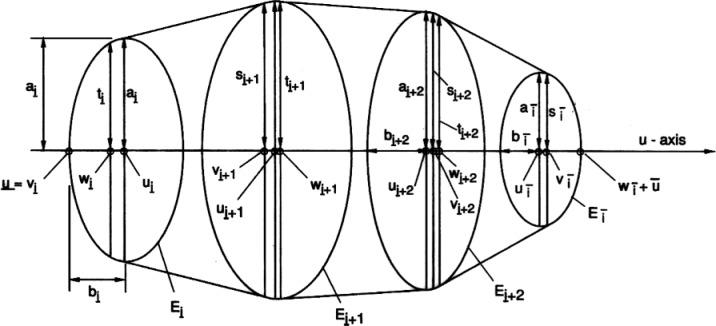
Convex envelope of elliptic projections of circular cross sections.

As a result, the approximate drag profile is described in the *u, y*-plane **P** by a concave function *y* = *p*(*u*) over a domain $\left[\underline{u},\bar{u}\right]$ with zero values at the endpoints. That domain is partitioned into segments in which the graph of this function is represented, in alternating fashion, either by a straight line or by an elliptical arc (Fig. 3). The first and last segments are elliptical segments. Thus $\underline{u}$ is the left minor axis point of ellipse **E**_i_, whereas $\bar{u}$ is the right minor axis point of ellipse **E***_i_*. This yields for the endpoints of the approximating drag profile:

$$\underline{u}=u_{i}-b_{i},\;\bar{u}=u_{i}+b_{i}.$$

We denote the break points for the partition into straight and elliptic segments by:

$$\underline{u}=v_{\underline{i}}\leq w_{\underline{i}}<\ldots <v_{i}\leq w_{i}<\ldots <v_{\bar{i}}\leq w_{\bar{i}}=\bar{u}$$

The possibility that $v_{\underline{i}}=w_{\underline{i}}$ and $v_{\bar{i}}=w_{\bar{i}}$ reflects the fact that the ellipses $E_{\underline{i}}$ and $E_{\bar{i}}$ at the beginning and the end of the approximate drag area may be just single points. The corresponding segments are then of zero length.

The following quantities, which are independent of the angle of attack *θ*, play a role in determining expressions for breakpoints *v*_i_, *w*_i_:

$$\Delta_{i}=\frac{a_{i+1}-a_{i}}{x_{i+1}-x_{1}}$$

With these quantities, we have:

$$\begin{array}{l} v_{i}=u_{i}-b_{i}\frac{\Delta_{i-1}}{\tan\theta },\;\underline{i}<i\leq \bar{i},\\ w_{i}=u_{i}-b_{i}\frac{\Delta_{i-1}}{\tan\theta },\;\underline{i}<i\leq \bar{i} \end{array}$$

For the corresponding *y*-coordinates we find using the equation of ellipse **E***_i_*:

$$s_{i}=p(v_{i})=a_{i}\rho_{i-1},\;t_{i}=p(w_{i})=a_{i}\rho_{i}$$

where

$$\rho_{i}=\sqrt{1-{\left(\frac{\Delta_{i}}{\tan\theta }\right)}^{2}}.$$

In general, the elliptical arcs will be much smaller than the straight line segments except for the first and last elliptical arcs which may well be longer. We therefore recommend replacing all intermediate elliptic arcs by their chords while keeping elliptic arcs at the ends. The corresponding calculations of drag area and drag center can be carried out in closed form.

The total approximate size A_TOT_ of the drag area A_DRAG_ is the sum

$$A_{\mathrm{DRAG}}\approx A_{\mathrm{TOT}}=A(v_{\underline{i}},w_{\underline{i}})+\ldots +A(w_{i-1},v_{i})+A(v_{\bar{i}},w_{\bar{i}})$$

of the areas of the vertical “strips,” above and below the *u*-axis, into which the drag profile has been divided. All such strips with the possible exception of the first and last ones are trapezoids that are symmetric about the *u*-axis. Those strips which were originally bounded by elliptical arcs have area

$$A(v_{i},w_{i})=(w_{i}-v_{i})(s_{i}+t_{i})\frac{\Delta_{i-1}-\Delta_{i}}{\tan\theta }$$

for $\underline{i}\leq i\leq \bar{i}$. For those strips which were trapezoidal from the beginning, we find

$$A(w_{i},v_{i+1})=(v_{i+1}-w_{i})(t_{i}+s_{i+1})$$

for $\underline{i}\leq i\leq \bar{i}$. The two elliptic end-strips have areas

$$\begin{array}{c} A(v_{i},w_{i})=a_{i}b_{i}\left(\sin^{-1}\rho_{i}-\rho_{i}\frac{\Delta_{i}}{\tan\theta }\right)\\ A(v_{i},w_{i})=a_{i}b_{i}\left(\sin^{-1}\rho_{i-1}-\rho_{i}\frac{\Delta_{i}-1}{\tan\theta }\right) \end{array}$$

Due to the symmetry of the drag area about the *u*-axis, the drag center lies on the *u*-axis, and its *y*-coordinate *y*_DRAG_ is therefore zero. Let *c*(*v*_i_,*w*_i_), *c*(*w*_i_, *v*_i+1_) denote the centers of area moment of their respective strips. Defining

$$\begin{array}{l} C_{\mathrm{TOT}}=A(v_{i},w_{i})c(v_{\underline{i}},w_{i})+\ldots A(v_{i},w_{i})c(v_{i},w_{i})\\ \;+A(w_{i},v_{i+1})c(w_{i},v_{i+1})+\ldots +A(v_{i},w_{i})c(v_{i},w_{i}) \end{array}$$

we find approximately for the *u*-coordinate *u*_DRAG_ of the drag center,

$$u_{\mathrm{DRAG}}\approx \frac{C_{\mathrm{TOT}}}{A_{\mathrm{TOT}}}.$$

Again we consider the two kinds of trapezoidal strips. For those that were originally elliptical, we find for $\underline{i}\leq i\leq \bar{i}$:

$$c(v_{i},w_{i})=\left(\frac{2s_{i}+t_{i}}{3(s_{i}+t_{i})}v_{i}+\frac{s_{i}+2t_{i}}{3s_{i}+t_{i}}w_{i}\right)$$

for the strips bounded by common tangents, we have for $\underline{i}\leq i\leq \bar{i}$.

$$c(w_{i},v_{i+1})=\left(\frac{2t_{i}+s_{i+1}}{3(t_{i}+s_{i+1})}w_{i}+\frac{t_{i}+2s_{i+1}}{3t_{i}+s_{i+1}}v_{i+1}\right).$$

The two elliptic end-strips, finally, contribute as follows:

$$\begin{array}{l} c(v_{\underline{i}},w_{\underline{i}})=u_{\underline{i}}-\frac{2a_{\underline{i}}b_{\underline{i}}^{2}\rho_{\underline{i}}^{3}}{3A(v_{\underline{i}},w_{\underline{i}})},\\ c(v_{i},w_{i})=u_{i}-\frac{2a_{i}b_{i}^{2}\rho_{i}^{3}}{3A(v_{i},w_{i})}. \end{array}$$

## 5. Selecting Cross Sections

The next question concerns the selection of the cross sections, that is, of the locations of *x_i_*. Clearly, we want to include the end points of the contour curve: $x_{1}=\underline{x}$, $x_{n}=\bar{x}$. If a cylindrical middle section is present, then cross sections are needed only at the beginning ${\underline{x}}_{\mathrm{HORIZ}}$ and the end ${\bar{x}}_{\mathrm{HORIZ}}$ of that section. Indeed, intermediate cross sections in the cylindrical portion clearly do not contribute to the convex envelope.

**Fig. 4:**
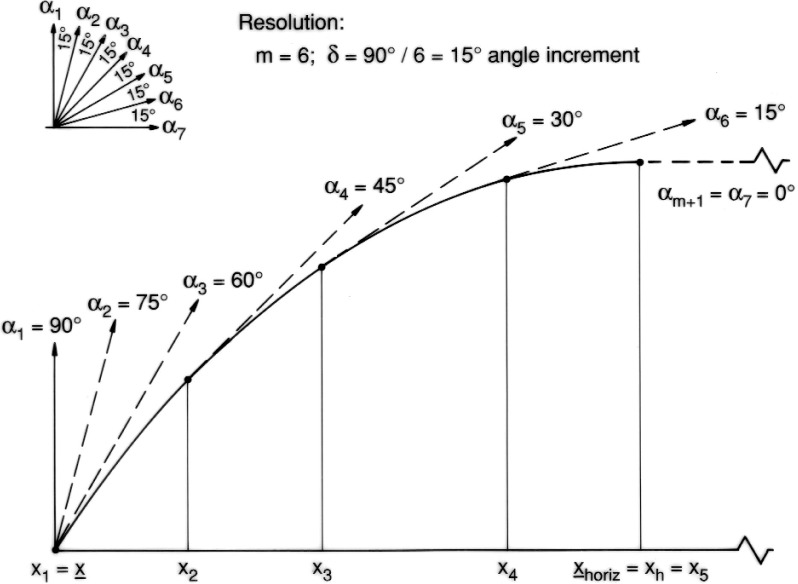
Ascending portion of contour subdivided by equal increments of tangential angle.

As to selecting cross sections in the ascending or descending portions, the density of the cross sections should increase with the curvature of the contour function. A straightforward way to achieve this is to select according to equal increments *δ* of tangential angles as follows. For a positive integer *m* we consider the angles (see Fig. 4).

$$a_{k}=90{}^{\circ}-(k-1)\delta \;\mathrm{for}\;k=1,2,\ldots ,2m+1$$

with angle increment

$$\delta =\frac{90{}^{\circ}}{m}.$$

Then we select those *x*-coordinates for which the contour function *z* = *r*(*x*) has the prescribed tangential angles *a_k_*, and place the cross sections there.

Determining the *x*-coordinates at which the given angles *a_k_* are assumed is by a straightforward bipartition scheme based on the derivatives of the given parametric equations. In the case of a pointed left end the first angle *a_k_* to be considered is the largest one smaller or equal to the contour angle at that end. Analogous restrictions hold if the right end is pointed.

In addition, we link the choice of the integer *m*, which determines the above increment *δ* to the angle of attack *θ* so that it occurs among the tangential angles *a_K_*. In that case, both ends $\underline{u}$, $\bar{u}$ of the approximate drag profile are exactly the ends of the actual drag profile. This is because the two *x*-coordinates at which tangents of angles ±*θ*, respectively, touch the graph of the contour function can be found among the selected coordinates *x_i_*. The ensuing conceptual simplification of the methodology, together with numerical advantages, justifies in our opinion the minor additional effort of placing the cross section coordinates according to equal increments of tangential angles. It usually has to be done only once for a series of angles of attack *θ*.

## 6. Analysis of the Space Shuttle External Tank and its Two Major Components

From engineering drawings [15] and oral communications [13] we derived measurements for a prototype external tank and its components, the LO2 and LH2 tanks.^2^ These measurements are indicated in Fig. 6. They do not refer to a particular tank—there are small differences between individual tanks—and the results given in this paper are not intended for any specific application but rather for demonstration of feasibility. All measurements are in inches from the base of the lightening rod at the forward tip of the external tank. The front is formed by the LO2 tank, whereas the LH2 tank, with two symmetrical rounded ends, forms the rear. These two component tanks each have cylindrical middle sections, which extend into the connecting cylindrical portion of the entire external tank. Parametric equations employed to describe the contours of these shells of revolution are also shown in Fig. 6.

Appendages such as the LO2 feedline and the forward and aft orbiter connection truss assemblies have not been included in these analyses due to the present limitation of being able to handle only one shell of revolution at a time. However, the above mentioned appendages are relatively small, with respect to the area of the overall tank, and should not greatly affect the results for orbital lifetime and station-keeping fuel calculations. Because the tank is modeled as a shell of revolution the results are insensitive to changes in roll angle about the axis of revolution.

**Fig. 5:**
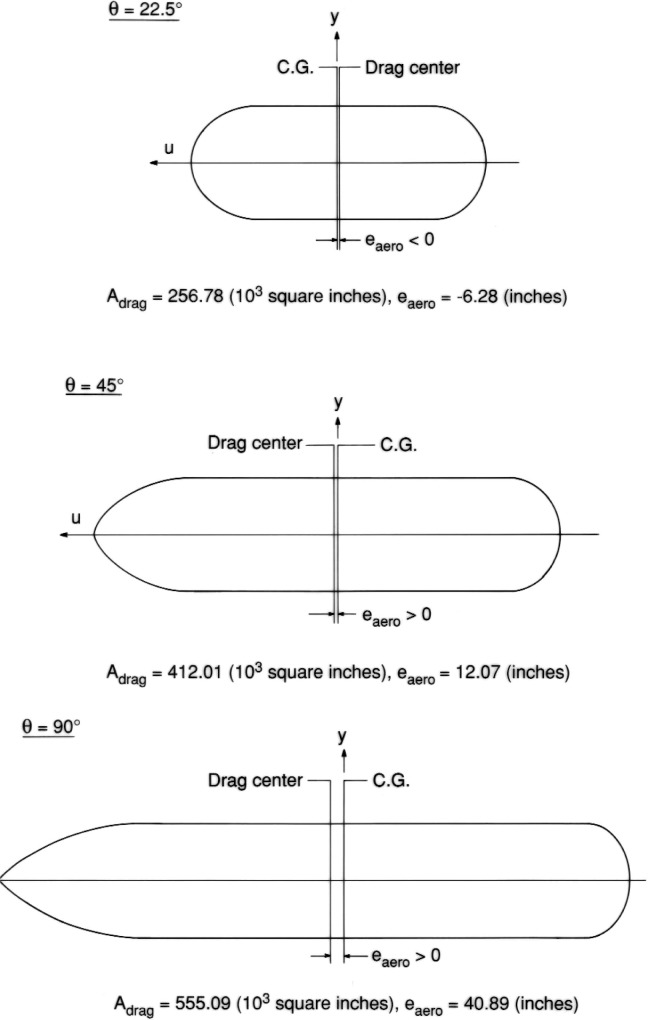
Drag profiles and eccentricities for the entire external tank.

**Fig. 6:**
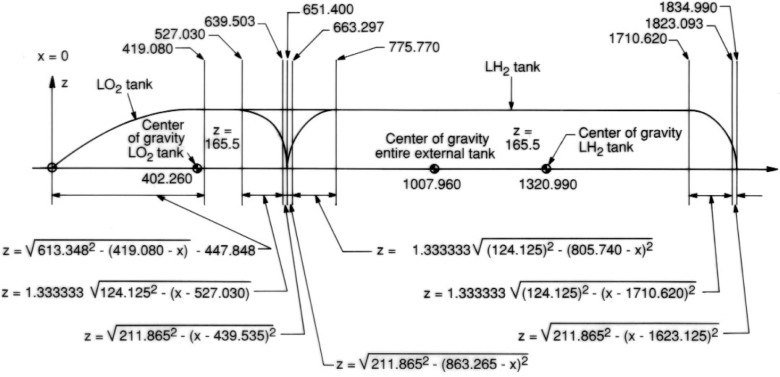
Measurements and parametric equations for external tanks.

Figure 5 presents, in tabular form, the results of an analysis of the whole external tank at three representative angles of attack. These drag profiles indicate that the algorithm is sufficiently robust to handle all possible angles of attach from 0° to 90°.

Some comments are in order concerning the general numerical performance of the method. The choice of the angle increment *δ* guides the selection of the cross sections: the smaller *δ*, the larger the number of cross sections. As one might expect, accuracy grows with the number of cross sections. Typically for discretization methods, however, there are limits beyond which further refinement of the discretization yields no improvement. This is due to the finite word-length of the computer—calculations are carried out in single precision—as well as subsidiary computations such as the determination of cross section location by prescribed tangential angles. For our calculations concerning the Space Shuttle external tank and its components, the limit appears to be reached for angle increment *δ* = 0.2°. With this limitation, the algorithm achieves a mathematical accuracy of five significant digits for the drag area, and three for the eccentricity. The latter is more sensitive because, for this particular application, the center of gravity and the drag center tend to be close.

In order to represent the drag area and the aerodynamic eccentricity of the external tank as a function of the angle of attack *θ*, the above quantities of interest have been evaluated at suitable intervals in preparation of fitting suitable regression equations. Certain inherent properties of these functions, however, should be preserved. Thus, the value A_0_ of the drag area for *θ* = 0° is exactly the area of the circular cross section of maximum diameter. That known value should be precisely reproduced. In addition, the drag area function has a maximum with horizontal tangent at *θ* = 90°. Analogously, the aerodynamic eccentricity vanishes for *θ* = 0° and also has a horizontal tangent at *θ* = 90°. These two considerations suggest fits of the functional forms A_0_ + **P**(sin *θ*) sin *θ* and **Q**(sin *θ*) sin *θ* respectively, with polynomials P and **Q** determined by the least squares regressions:

$$P(\sin\theta )\approx \frac{A_{\mathrm{DRAG}}-A_{0}}{\sin\theta },\;Q(\sin\theta )\approx \frac{e_{\mathrm{DRAG}}}{\sin\theta }.$$

Such regression equations were obtained for the entire external tank as well as its two constituent tanks. They were based in each case on 18 evaluations with *θ* ranging from 5° to 90° at intervals of 5°. For these evaluations, the cross sections were placed according to an angle increment *δ* = 0.25°. The equation for drag area as a function of angle of attack, *θ*, for the entire tanks is given by:

$$\begin{array}{l} A_{\mathrm{DRAG}}=86,049+425,\;680.2\;\sin\theta \\ \;+62,195.0\;\sin^{2}\theta -18,409.5\;\sin^{3}\theta . \end{array}$$

The units for A_DRAG_ are square inches. Similarly, the aerodynamic eccentricity can be represented as follows:

$$\begin{array}{l} e_{\mathrm{DRAG}}=-55.805\sin\theta +94.\;439\;\sin^{2}\theta \\ \;+35,210\;\sin^{3}\theta -32.966\;\sin^{4}\theta . \end{array}$$

The units for e_AERO_ are inches. Both equations are valid for 0° < *θ* ≤ 180°.

**Fig. 7:**
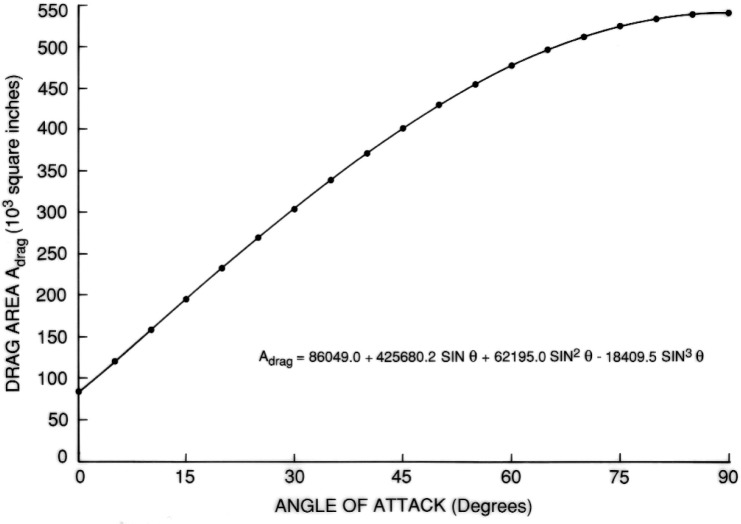
Equation for drag area of entire tank.

**Fig. 8:**
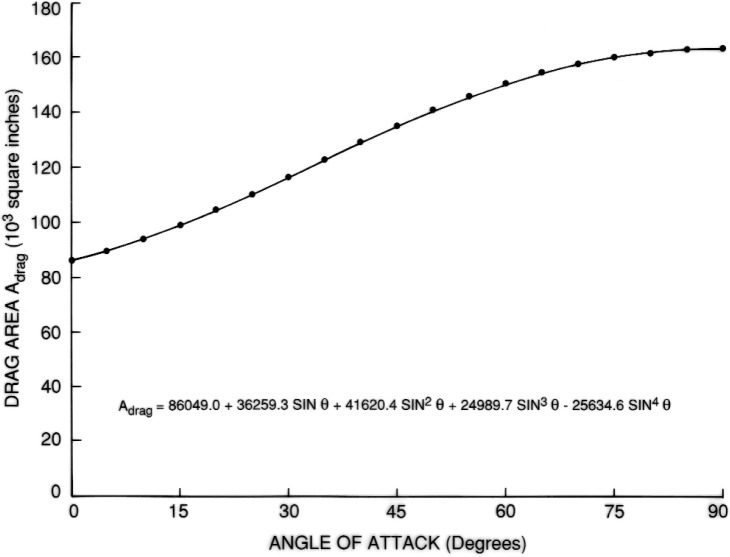
Equation for drag area of LO2 tank.

**Fig. 9:**
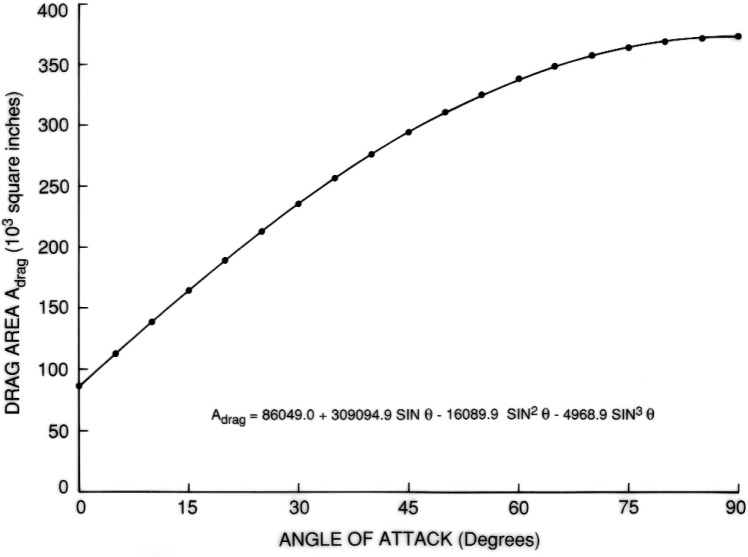
Equation for drag area of LH2 tank.

**Fig. 10:**
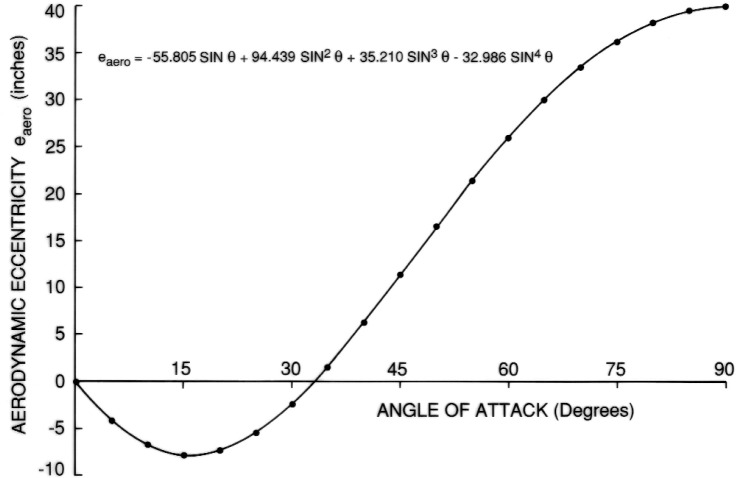
Equation for eccentricity of entire tank.

**Fig. 11:**
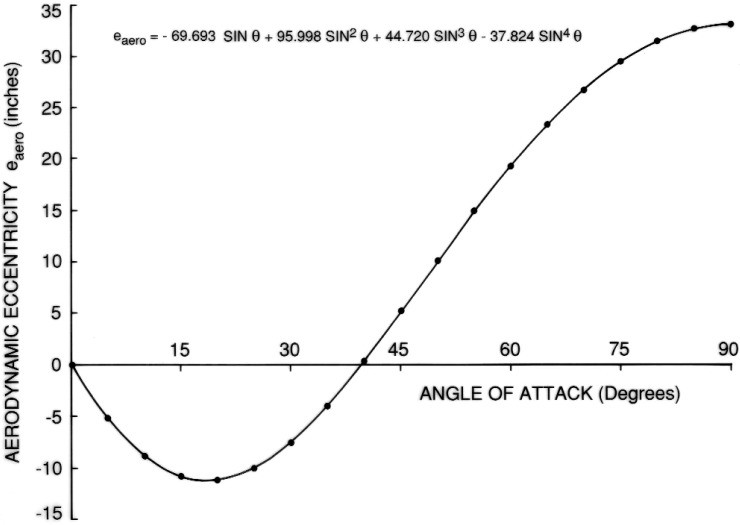
Equation for eccentricity of LO2 tank.

**Fig. 12:**
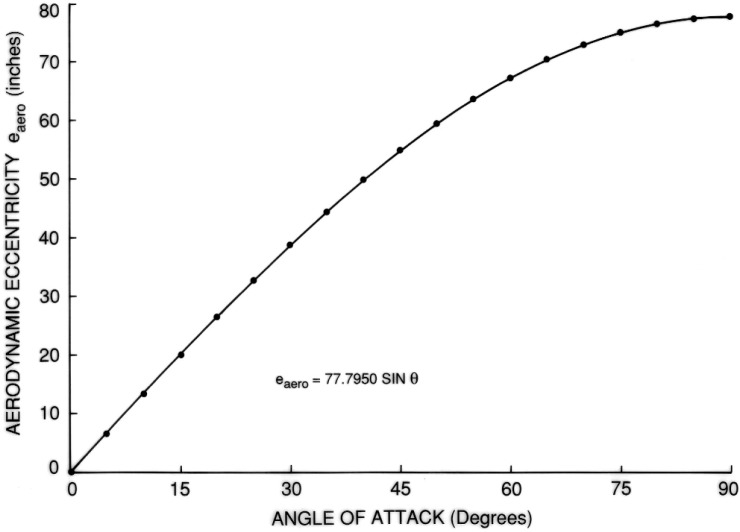
Equation for eccentricity of LH2 tank.

The results are shown in Figs. 7–12, which also contain plots of curves, representing regression equations, against data points, representing evaluations. The plots indicate agreement within at least two significant digits. All regression equations are valid for

0°<θ≤180°

where *θ* denotes the angle of attack. Should a value of *θ* between 180° and 360° be specified, then it has to be replaced by (360°–*θ*).

Note that, for the symmetric LH2 tank, the eccentricity is given as a pure sine wave. This is not an approximation: if the shell of rotation has a center of symmetry, then the eccentricity of that shell as a function of the angle of attack is exactly of the form e sin *θ* for any position of the center of gravity along the axis of revolution.

## 7. Derivation of Breakout Formulas

We will now address the derivation of the expressions used above for the contour breakpoints *v_i_* and *w_i_*. We will prove that for $\underline{i}\leq i\leq \bar{i}$:

$$w_{i}=u_{i}-b_{i}\left(\frac{a_{i+1}-a_{i}}{u_{i+1}-u_{i}}\right)\cos\theta =u_{i}-b_{i}\frac{\Delta }{\tan\theta }$$

where

$$\Delta_{i}=\frac{a_{i+1}-a_{i}}{x_{i+1}-x_{i}}.$$

Here *a_i_* and *a_i_*_+1_ are the major axes and *b_i_* and *b_i_*_+1_ the minor ones, and *u_i_* = *x_i_* sin*θ*, *u_i_*_+1_ = *x_i_*_+1_ sin*θ* the *u*-coordinates of the centers of the two consecutive ellipses **E***_i_* and **E***_i_*_+1_.

There are, in general, two pairs of common tangents. Here we are only concerned with those common tangents that leave both ellipses on the same side. Such tangents exist if one ellipse does not contain the other. If both ellipses are of the same size, *a_i_* = *a_i_*_+1_, then the aforementioned formulas are obviously correct. Thus we are left with the case *a_i_* ≠ *a_i_*_+1_. In this case there exists a center of similarity (*u*_SIM_, *y*_SIM_) = (*u*_SIM_,0) on the *u*-axis such that the scale to which the ellipses are drawn is proportional to their distance from that center. For the major axes *a_i_*, *a_i_*_+1_ that implies:

$$\frac{a_{i+1}}{a_{i}}=\frac{u_{i+1}-u_{\mathrm{SIM}}}{u_{i}-u_{\mathrm{SIM}}}.$$

From this

$$\begin{array}{l} u_{\mathrm{SIM}}=\frac{a_{i+1}u_{i}-a_{i}u_{i+1}}{a_{i+1}-a_{i}},\\ u_{\mathrm{SIM}}-u_{i}=-a_{i}\left(\frac{u_{i+1}-u_{i}}{a_{i+1}-a_{i}}\right)=-\frac{a_{i}\sin\theta }{\Delta_{i}}. \end{array}$$

It is also clear that any straight line connecting similar points of the two ellipses, respectively, must pass through the center of similarity. The points of equal slope above—and also those below–the *u*-axis on each ellipse are such similar points. The two common tangents, in particular, intersect therefore at that center. It follows that the vertical line *u*-**w***_i_*, which connects the points of contact of those tangents, is the polar of the center of similarity with respect to ellipse **E***_i_* and must thus agree with the line described by the well-known formula for the polar:

$$\frac{\left(u-u_{i})(u_{\mathrm{SIM}}-u_{i}\right)}{b_{i}^{2}}+\frac{yy_{\mathrm{SIM}}}{a_{i}^{2}}=\frac{\left(u-u_{i})(u_{\mathrm{SIM}}-u_{i}\right)}{b_{i}^{2}}=1.$$

Thus, and since *b_i_* = *a_i_* cos*θ*,

$$w_{i}=u_{i}+\frac{b_{i}^{2}}{u_{\mathrm{SIM}}-u_{i}}=u_{i}+\frac{b_{i}a_{i}\cos\theta }{u_{\mathrm{SIM}}-u_{i}}.$$

An expression for u_SIM_–*u_i_* has just been derived and upon substitution yields the desired expression for *w_i_*.

## 8. Concluding Remarks (1989)

The numerical method presented in this paper has the advantage of providing a self-contained subroutine which can be inserted directly into an orbital lifetime and station keeping analysis program. The method extends naturally to shells of revolution which are not members of the category treated in this paper. We are planning to extend software capabilities accordingly to permit straight line segments in the contour other than the horizontal one, and to relax the requirement for unique tangents at each point along the shell. Beyond this we feel that even the requirement of convexity can be relaxed and that small appendages and their shielding effects can be treated as long as these appendages are shells of revolution themselves with axes parallel to the axis of the main body.

### Post Scriptum

A slightly extended version of this paper was published in the *Journal of Aerospace Engineering* [16] in 1991. By that time a number of follow-on studies had been performed at NIST and elsewhere relating to the stabilization of the external tank in LEO. Importantly, propulsion and orbital lifetime calculations (accounting for atmospheric drag using the procedures developed in this paper) were conducted at NIST [17, 18] in 1990. Tentative engineering designs were developed for an aft propulsion and guidance package that could be strapped to the stern of the E-tank and would permit autonomous testing of the ability to guide the E-tank, post-MECO, and to store it in LEO. The following summary, from reference [17], attests to just how little effort would have been involved to do so:

“… it was calculated that the space shuttle external tank can be boosted to a short term stable orbit following standard MECO separation from the shuttle orbiter, and without any direct interaction nor detriment to orbiter performance. An exterior propulsion package for the external tank equipped with a minimum thrust capacity of 4,448 N (1,000 lbf), a propellant mass of 500 kg (1,100 lbm), and an ISP of 400 s appears sufficient to achieve an initial time in orbit of nearly two days under solar maximum conditions, provided the burn is made at initial apogee and the angle of attack is maintained near zero degrees by an onboard attitude control system. It is assumed that additional velocity change burns will take place following the initial burn which will place the tank in a circular orbit between 400 km to 500 km altitude for long term storage. Initial estimates of the total fuel required to achieve a 500 km circular storage orbit come to 2,059 kg (4,537 lbm) based upon Hohmann transfer theory following the initial apogee burn. All calculations assumed 5,000 lbm (2,270 kg) of residual cryogens in the external tank following MECO as *deadweight*. Recovery and use of these propellants by the exterior propulsion package would lead to a dramatic increase in the time in orbit above the values reported in this paper.”

Thus, an expenditure of two metric tons of propellant and the addition of a likely 2–400 kg of guidance and propulsion structure in the aft control package would have placed in long term orbit 32 metric tons of high grade aerospace structure with a redundant, pressurizable internal volume of 2,069 m^3^—more than 10 times that of the International Space Station in 2004—that could be put to use by industry for commercial labs, fuel depots, construction shacks, hotels and the like. In today’s dollars this would have equated to an investment of $69M (if the aft propulsion package was paid for at the going rate per kilogram to LEO) to place a $960M asset in orbit (32 metric tons of E-tank x $30K per kg), an investment leveraging of 14 to 1. In actuality, the cost of the aft propulsion package would have amounted to only the engineering development costs and the integration of off-shelf technology for an aggregate outfitting expense of less than $10M per tank and possibly as low as $2M to $5M per tank after the initial concept had been demonstrated. The extra mass does not need to be considered in this particular case.

One might conclude in light of the above arguments that action would have been taken to capitalize on these concepts. This was not the case, however, and the final part of the story is worth telling. On the basis of the calculations in [17, 18] and with the encouragement of industry, NIST developed a proposal to conduct an on-orbit test to stabilize the E-tank and ultimately to cause it to re-enter in a controlled fashion following conclusion of the test flight. It was proposed, furthermore, to have a NIST scientist be the payload specialist for that mission. The proposal was examined by industry (The External Tanks Corporation as well as the Martin-Marietta division that manufactured the tanks) and had the support of the National Corporation for Atmospheric Research (NCAR) and the scores of universities that it represented. With that blessing the proposal made its way up the management chain until it became apparent at the Commerce level that there was a problem. The problem had nothing to do with the validity of the engineering. Simply put: the placement of such a large, pressurized, human-rated facility in LEO was viewed as competition to the International Space Station. Contracting companies, recognizing that their contract to continue production of the E-tank might be in jeopardy, ceased support for alternative uses of the vessel. The political hot potato was quietly dropped. America would tolerate only one “space station” project. Today, the entrepreneurial space community has written off the possibility of using E-tanks in orbit—a casualty of their political baggage—and is looking at other alternatives (mainly inflatable structures that can be launched from standard ELVs) for placement of large, lightweight facilities in orbit that they, not government, will control.

## Footnotes

1 Most of the material in this paper first appeared in “Space Manufacturing 7: Space Resources to Improve Life on Earth,” Proceedings of the Ninth Princeton/AIAA/SSI Conference, May 10–13, 1989.

2 Both references listed, as well as NASA manufacturing and engineering drawings for the External Tank, utilize units of inches for length measurement. While it is NIST standard practice in 2004 to use SI units, for the purposes of this historical document we have retained the system of units originally presented in the paper in 1989 for the remaining figures. SI readers will note that 1 inch = 25.4 mm and 1 in^2^ = 6.4516 cm^2^.
